# The Impact of Educational Resources and Perceived Preparedness on Medical Education Performance

**DOI:** 10.1007/s40670-021-01306-x

**Published:** 2021-05-26

**Authors:** Justin Bauzon, Amalie Alver, Vishvaas Ravikumar, Adrian Devera, Tatiana Mikhael, Rafae Nauman, Edward Simanton

**Affiliations:** 1 Kirk Kerkorian School of Medicine at the University of Nevada Las Vegas (UNLV), Las Vegas, NV 89154 USA; 2 Department of Education, Kirk Kerkorian School of Medicine at UNLV, Las Vegas, NV 89154 USA

**Keywords:** Medical student resources, Exam performance, Confidence

## Abstract

**Introduction:**

Undergraduate medical education has evolved necessarily with the increasing utilization of technology and the availability of ancillary resources developed for medical students. However, medical educational resources are expensive and there have been few studies validating these resources for their ability to significantly modify student exam performance.

**Methods:**

A post-exam survey was devised to evaluate medical students for resource usage, student-perceived preparedness, and exam performance.

**Results:**

Students who felt more prepared for exams performed better than students who felt less prepared (*p* = .017). Students who watched didactic lectures online and those who utilized peer-to-peer tutoring outperformed students who did not use these resources (*p* = .035, *p* = .008). Analyses of the data show that none of the purchased resources utilized significantly improved student exam performance. The majority of students used between six and eight resources for exam preparation. There may be a slightly negative association with the quantity of resources used and exam scores (*p* = .18).

**Discussion:**

Contrary to traditional confidence studies that correlate overconfidence with underperformance, medical students who reported feeling more prepared for exams performed better than students who felt less prepared.

**Conclusion:**

Medical students may have a more complete grasp of their knowledge base and deficits, which may enable a more accurate match between exam expectations and academic performance. This post-exam survey method can be customized and applied to evaluate resource utility as it pertains to specific undergraduate medical education curricula at individual institutions.

## Introduction

Medical school curricula and available student resources have drastically evolved alongside advancements in technology and the internet [1, 2]. The medical students of today are not the medical students of their predecessors. They must constantly review new information as it becomes available. With the current rate at which medical knowledge is updated, it is crucial to ensure that future physicians are comfortable synthesizing and applying new evidence as it unfolds. This has been observed in the recent transition to a “competency-based” approach where students are taught to seek out and apply new information readily [3]. In the same token, the exponential growth in availability of online medical educational resources over the past decade has placed the onus primarily on medical students to ascertain which is the most effective method of knowledge acquisition outside of their formal curriculums [2, 4].

Scheduled progress exams serve to assess a student’s breadth of knowledge in the preclinical setting. It is thus of great interest for medical educators and students alike to identify which modifiable factors will result in the highest utility for exam preparation. Prior studies have found a negative correlation when comparing student overconfidence with examination success [5]. Determining which resources instill a favorable level of confidence while still best preparing students is important for developing a curriculum that both meets the expectations of students and fulfills the goals of the educators [6]. It has also been suggested that the total number of resources used for preparation may have an effect on exam score [7]. The present study examines how post-exam preparedness relates to test performance for first-year students at a new allopathic school. The most commonly utilized preclinical resources used for exam preparation were evaluated for how they influenced test performance and student confidence on exams. We lastly posit that increasing the number of resources utilized will lead to poorer performance outcomes.

## Materials and Methods

Our study evaluates the resource usage of medical school students in the preclinical phase of a newly formed medical school. Our curriculum utilizes an organ systems-based approach to exams using the National Board of Medical Education’s (NBME®) Customized Assessment Services. This tool allows educators to develop exams by selecting from a large pool of NBME® questions with content specific to the current curricular testing block. Tests are timed and administered as biweekly progress exams to a cohort of 60 students. Scoring is measured on a 100-point scale, and educator-facing features allow for the analysis of how examinees compare to the performance of medical schools throughout the USA.

We developed an anonymous post-exam survey that assessed for the following components:

- A “Preparedness Score” (PS), which asked students how prepared they felt about their exam performance on a scale of 1 to 10;
- A list of potential resources students may have used to prepare, which included:
- Formal curriculum resources that are recommended in the institution’s preclinical syllabus, which included in-person lectures, online recorded lectures, in-person anatomy sessions, and assigned textbook readings;
- Extracurricular resources outside of the syllabus, which included peer-to-peer tutoring sessions with above-level medical students; online medical education video-based supplemental curricula (Boards & Beyond®, Pathoma™); practice question banks (Kaplan™ Step 1 QBank, AMBOSS©); visual learning platforms (SketchyMedical©); flashcard review programs (Anki); and study preparation books (First Aid for the USMLE® Step 1);
- A “Resource-Specific Score” (RSS; scale of 1 to 10) that ascertained how well students felt each utilized resource helped prepare them for each exam;
- A free response comment section for additional written feedback.

**Fig. 1:**
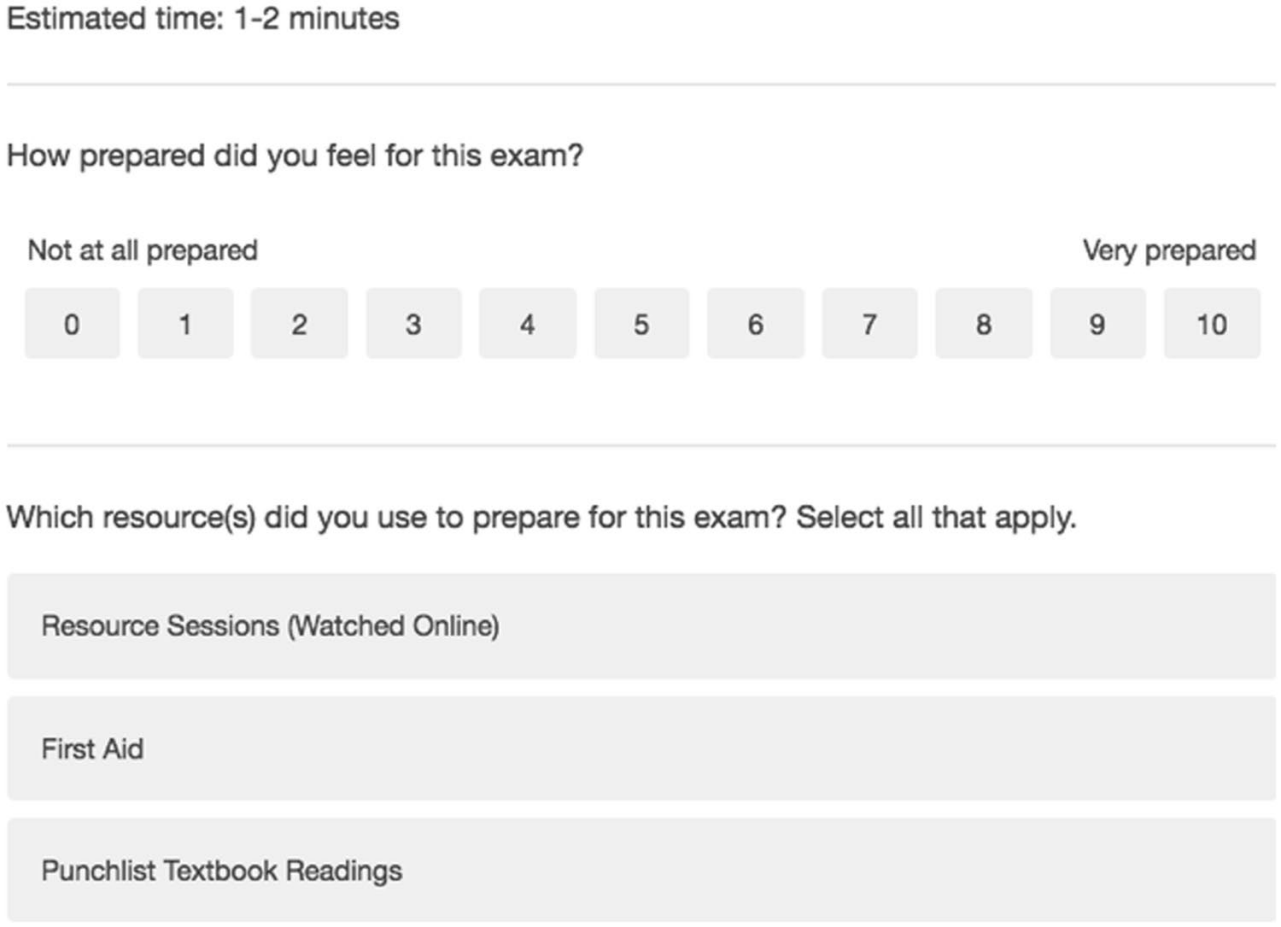
A representative view of post-exam survey

Figure 1 shows a portion of the survey administered to students. This survey was voluntarily completed and administered over eight systems-based NBME® examinations. All participants of this study were informed of survey administration prior to their exams and were given the option to opt out of participation. Surveys were conducted after completion of each exam and before exam scores (ES) were released. Examinees submitted survey results with an anonymized testing ID number. An exam proctor blinded from student responses to the survey paired the final ES with their associated survey results; only fully completed surveys with a relevant testing ID number were included in this study. Data analysis included a linear regression of ES and PS using the Pearson correlation coefficient, an unpaired *t* test to compare the ES/PS of each resource utilized versus its non-utilization, and the regression analysis of the summative number of resources used and overall ES/PS.

The post-examination questionnaire utilized in this study is included as an online supplement file to provide a model for faculty who wish to incorporate similar studies at their institutions.

## Results

There were 161 survey responses collected among eight separately administered examinations. Of the 60 students for each exam session, between 15 and 55% responded to the post-exam survey, with an average response rate of 34% per exam. The mean ES among respondents was 78.7 (ranging between 71.1 and 85.2 across exams), compared to 81.7 across all examinees. The average reported PS was 6.0 (with a range of 4.7 to 7.6 across all exams). Table 1 summarizes the results of respondee’s ES, PS, and overall class ES averages among all exams.

**Table 1:** Exam score and preparedness score averages across eight exams

	Average exam score (survey respondees)	Average exam score (overall class)	Mean preparedness score
Exam #1	85.2	84.5	6.97
Exam #2	82.3	82.0	4.71
Exam #3	83.4	81.9	5.66
Exam #4	71.1	79.0	6.64
Exam #5	73.7	81.2	5.33
Exam #6	72.5	83.6	7.67
Exam #7	74.2	82.8	6.17
Exam #8	73.3	78.7	5.13
All exams	78.7	81.7	6.97

### Perception of Test Performance Versus Exam Outcome

**Fig. 2:**
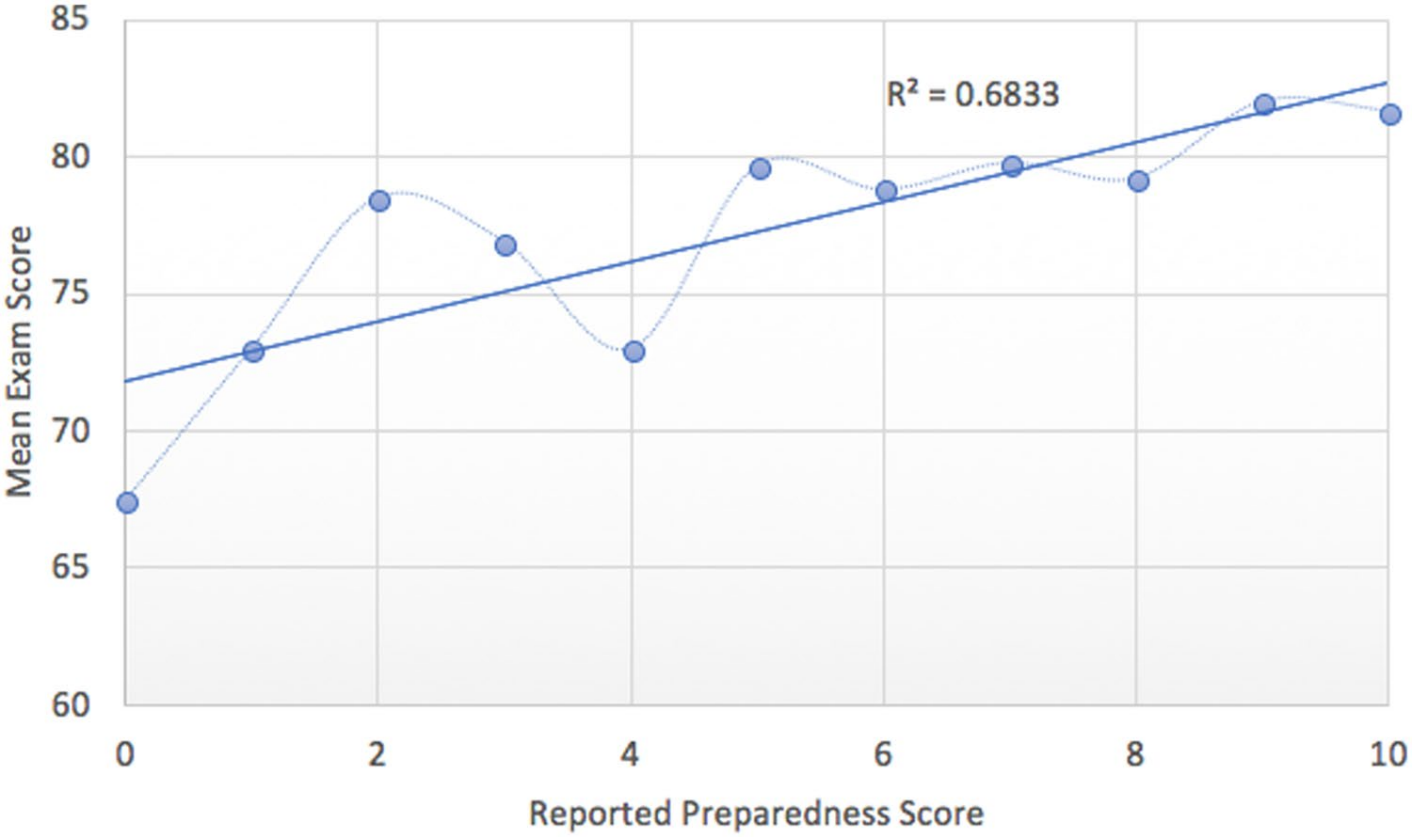
Average exam score results when compared to the preparedness score of respondents

We used an alpha level of 0.05 for all statistical tests. Among survey respondents, the reported PS and mean ES results were observed to be strongly positively correlated, *r*(9) = 0.83, *p* = 0.017. There was a minimum average ES of 67.5 and maximum of 82.0 (for a PS of 0 and 9, respectively). Figure 2 shows the correlation between mean ES and PS.

### Relationship Between Specific Resources Utilized and Exam Performance

Table 2 summarizes the individual use of specific resources for exam preparation and the average ES, PS, and RSS. Examinees who attended online lectures were found to have a 4% increase in ES compared to non-attendees (*p* = 0.035); those who utilized our curriculum’s peer-to-peer tutoring were found to have a 7% improvement (*p* = 0.008). When using Kaplan’s QBank to prepare for exams, there was somewhat of an improvement in overall exam performance (ES of 79 vs 76, *p* = 0.08). Six of the resources utilized were associated with a score higher than the overall mean ES: in-person and online lecture attendance, assigned textbook readings, Kaplan’s QBank, tutoring sessions, and Pathoma. Non-use of Boards & Beyond and AMBOSS ES were slightly higher than 78.7.

**Table 2:** Outcomes of exam resource utilization and non-utilization on overall exam performance, perceived preparedness, and resource-specific scores

	Mean ES with resource/without resource	*P* value (confidence interval)	Mean PS with resource/without resource	*P* value (confidence interval)	Mean RSS
Attended in-person lectures	79.0/78.4	.62 (− 1.9–3.2)	5.8/6.2	.30 (− 1.1–0.33)	6.6
Attended online lectures	**81.3/78.0**	**.035 (0.24**–**6.5)**	5.9/6.0	.81 (− 0.96–0.75)	5.1
Assigned textbook readings	78.8/78.5	.78 (− 2.2–2.9)	5.8/6.2	.30 (− 1.1–0.33)	7.8
Anatomy sessions	72.1/74.4	.16 (− 5.4–0.92)	5.9/6.3	.40 (− 1.5–0.58)	7.5
Kaplan QBank	79.2/76.3	.08 (− 0.35–6.2)	6.1/5.7	.42 (− 0.52–1.2)	7.7
AMBOSS	78.6/78.8	.88 (− 2.8–2.4)	6.0/6.0	.86 (− 0.75–0.63)	7.6
Boards & Beyond	78.5/81.2	.31 (− 8.0–2.6)	6.0/5.6	.56 (− 1.0–1.9)	7.8
First Aid	78.5/79.3	.66 (− 4.2–2.7)	6.0/5.9	.78 (− 0.81–1.1)	8.3
Sketchy	78.7/78.6	.97 (− 2.5–2.6)	6.2/5.8	.24 (− 0.28–1.1)	8.3
Tutoring sessions	**83.3/78.0**	**.008 (1.4**–**9.2)**	5.8/6.0	.67 (− 1.3–0.84)	8.1
Pathoma	78.9/78.3	.63 (− 2.0–3.3)	5.8/6.2	.26 (− 1.1–0.30)	7.9
Anki	76.0/76.6	.80 (− 5.1–3.9)	6.1/5.1	.11 (− 0.21–2.1)	8.1

*ES* exam score, *PS* preparedness score, *RSS* resource-specific score. **Bold** values are statistically significant.

When compared to the overall mean PS, survey respondents were found to have reported a marginal increase in perceived preparedness when using Kaplan QBank, Sketchy, and Anki. Conversely, PS were slightly higher when not using in-person lecture attendance, assigned textbooks, anatomy sessions, and Pathoma. No significant differences in PS were observed when comparing any individual resource use to non-use.

On average, examinees rated the specific resources higher than their overall perceived preparedness for an exam (i.e., RSS > PS), with the exception of attending online lectures. The resources with the highest reported RSS were First Aid, Sketchy, tutoring sessions, and Anki.

### Number of Resources Used and Exam Performance

**Fig. 3:**
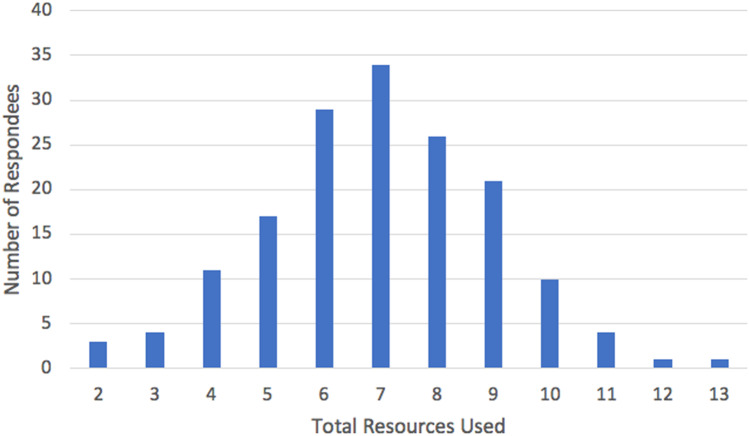
Graphical representation of the quantity of resources used by medical students

**Fig. 4:**
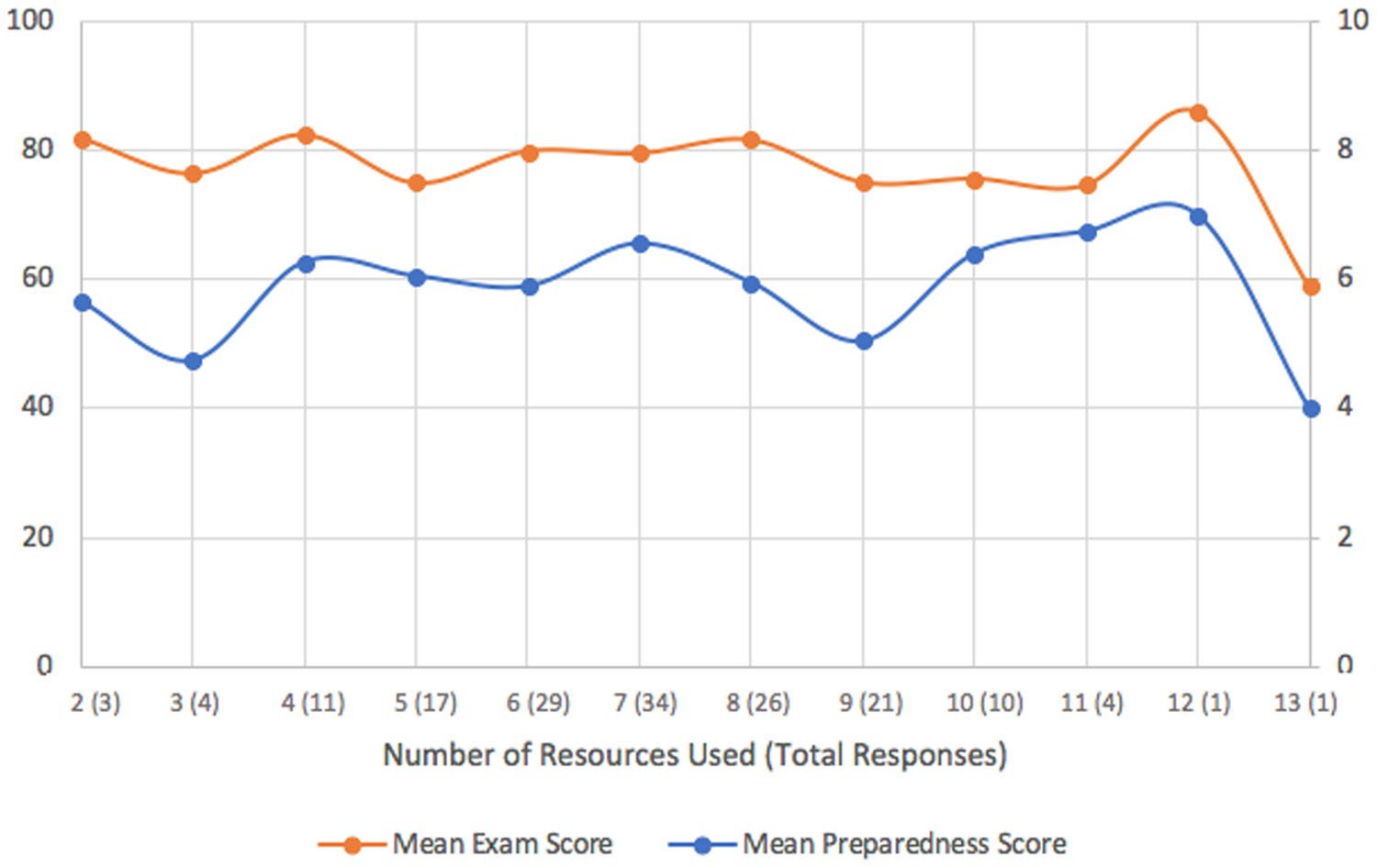
Number of resources utilized by examinees versus perceived and overall exam performance

The overall average number of resources used for each exam was seven and follows a normal distribution (standard deviation of 2.03; Fig. 3). Higher exam scores were moderately associated with less utilization of resources (Fig. 4), *r*(11) =  − 0.43, *p* = 0.18. There was no correlation between number of resources and average PS, *r*(11) = 0.05, *p* = 0.87. More than half of respondents (55%) used between six and eight resources for exam preparation. By far, the most utilized resources by examinees were Boards & Beyond, followed by First Aid and the Kaplan QBank (*n* = 151, 135, and 131, respectively).

## Discussion

Educational preparation resources available for undergraduate medical students have seen a substantial growth over the past decade [2, 4, 8]. In particular, advancements in technologies have paved the way for increased use of digital resources in medical education [1, 2, 9]. As a result, increased pressure is placed on educators and students alike to identify which resources are most effective for their respective curriculums and individual study habits. Determining if certain resources lead to scholastic improvements is critical to refining a developing medical curriculum, and our assessment of medical student resource utilization on academic results is an attempt to better understand the overall effectiveness of these resources.

The Dunning-Kruger effect describes the phenomenon where individuals with lower skill levels on a specific task overestimate their predicted performance [10]. This has concerning ramifications in the formal education setting, as students demonstrating suboptimal aptitude may underperform in test-taking scenarios. Low performance secondary to overconfidence has been attributed to resultant diminished exam preparation strategies, poor restudy decisions, and personal desires for higher scores biasing grade predictions [11–14]. Our study shows a significant positive correlation between student perceived preparedness and exam outcomes, which contrasts these traditional findings of overconfidence predicting worse academic performance. This suggests that confidence and testing outcomes may apply differently to medical students, who may have a greater self-awareness of their knowledge deficits post-matriculation into a medical program. This skill is essential to succeeding in medical education given the rigorous coursework and high performance expectations [15]. In contrast to performance in other academic settings [12–14], medical students may not be as susceptible to “the planning fallacy” in terms of overestimating how much dedicated study time is needed to obtain a high exam score [16]. As a result, post-exam expectations on how prepared students felt may more accurately represent their actual overall performance.

To our knowledge, there have been no studies that have compared undergraduate medical education resource usage and preclinical knowledge-based progress tests. We hypothesized that increasing the number of resources used would result in poorer outcomes as students would struggle with filtering and prioritizing the amount of available content. Our findings indicate a slightly negative correlation on exam performance with an increasing number of resources used. However, these results were not statistically significant, which aligns with other studies that did not find a major impact on exam performance with the number of resources used [17]. It is of note that reported resource numbers followed a normal distribution, implying that there is a comfortable range for most medical students.

A heavily debated aspect in undergraduate medical education is the utility of the traditional in-person didactic lectures. Attendance of in-class lectures has shown a marked decline over time and is a poor predictor for exam performance [18, 19]. When student attendance declines, faculty report decreased enthusiasm for their work and lower job satisfaction. Students cite many reasons for preferring online lectures, including efficiency and schedule flexibility [20]. Our results found the only resources which resulted in significant exam performance improvements were peer-to-peer tutoring and the use of lecture recordings. The latter of these is consistent with some previous studies [21], but it stands in contrast to other reported findings [22]. While our results show a clear positive association between the use of online lectures and exam performance, students rated the online lectures poorly on their perceived preparedness utility, with a mean reported RSS of 5.1. This may be due to the varying teaching style of educators at the institution, as rarely did any one individual teach across multiple organ systems.

Near-peer tutoring serves as an important adjunct to improve upon a student’s base medical knowledge. The results of our study showed a significant improvement in ES by students who utilized the school’s tutoring services. This may reflect the advantage of directly interfacing with peers who are more advanced in their medical education. Those who participate in these programs are afforded additional learning and revision opportunities in a low-stress, small group learning environment [23]. Participants receive the added benefit of obtaining insight and advice from their experienced peers [24]. There is unfortunately a dearth of published research on peer-assisted learning in medical education, an area that could benefit from future longitudinal studies [25].

None of the other resources in this study showed significant improvements in ES, which suggests for this specific curriculum that none are superior for improving student exam performance. Most students reported using between six to eight resources to prepare for exams, which make it difficult to evaluate the true utility of each resource in isolation. Indeed, certain resources may be synergistic when used in conjunction, or may have a proportionally greater impact when approaching certain topics compared to others [17]. For example, traditional textbook or lecture-based learning paired with expanded-retrieval platforms like Anki may lead to greater studying efficiency and improved overall outcomes [26]. It is possible that the students who performed better on exams in our study also utilized specific resources differently than those who had lower ES [27].

Medical students also report preferring resources that provide high-yield and easily accessible content [17]. Question banks have previously been reported to be the most frequently used resource by medical students. It has also been suggested that with the increased usage of these online question banks, medical schools should independently confirm the utility and validity of such resources with respect to their curriculums when offering them to students [6]. The results of our study did show a modest improvement in exam scores with the use of the Kaplan™ QBank. This may be partly due to the question bank format interfacing well with the longitudinal progress test format of our school’s curriculum [28]. Future studies could consider evaluating the use of question banks with a larger sample size to evaluate for truly significant impacts on exam performance.

There are a number of factors that may influence student resource choice, cost being one of the most important considerations [8, 17]. A recent study found the mean cost for study materials and exam fees during undergraduate medical education totaled nearly $7,500 on average [29]. The prohibitive expenses of medical school attendance coupled with growing student debts highlight the importance of being selective with effective, often non-budget-friendly resources [29]. With the increase in the number of resources, medical students have reported feeling overwhelmed by their myriad of options. Our students reported utilizing the school-provided resources much more frequently than independently purchased resources, which is reflected in the individual resource utilization sample size.

The survey used in this study provides a versatile method to allow institutions to identify potential gaps in their curriculum and determine which resources may most benefit their specific student populations. The increasing utilization of online learning by medical students prompts an investigation of how well resources are being used [30]. Our findings have helped inform our institution’s approach on how future student cohorts may best prepare for preclinical examinations, which includes selecting an ideal number of resources for study and utilization of near-peer sessions. However, we recognize that medical curricula, student compositions, and faculty backgrounds differ across academic institutions. To this effect, we provide the survey that may be customized to fit a specific institution’s needs. By administering this survey post-exam before students have access to their scores, student responses are not influenced by their overall exam performance. Anonymizing survey results also allows students to provide candid responses regarding resources used for study, including those developed for a school’s formal curriculum. Finally, the free response section offers an opportunity for students to provide their input on how faculty may improve future exams. The relative newness of our curriculum served as an opportunity to identify learning gaps early when preparing students for preclinical knowledge mastery. Evaluating which resources improve student performance may also help reduce the financial burden of the preclinical education years by providing students with curriculum-specific validated resources and may also allow faculty to determine which resources may best serve as supplements to their structured educational programs.

This study has limitations. Because ES were released to students at varying times after exams, some students may have taken the survey after seeing their score which could alter the student’s perception of their own preparedness and introduce response bias. We limited this occurrence by having the survey immediately accessible post-exam in the same room testing was administered. The survey was not mandatory, so respondents who felt more confident in their performance may have been more likely to participate. Fewer students completed the survey with each successive exam, so the sample size progressively decreased throughout the course of the study. Our institution’s curriculum is designed around organ systems, so resource usage throughout the year likely changed depending on the subject. The questionnaire was modified as the year progressed to include new resources used by students, which may have limited the data set for certain resources. Furthermore, our survey included an “Other” category for resources not directly included when the survey was conducted, which may lead to an over- or underrepresentation of a resource’s utility. Time spent with in-house learning support specialists (academic counselors, course directors) was a specific resource not considered in our initial study design, which may have impacted overall confidence and academic performance and warrants future study.

## Conclusions

Contrary to traditional norms widely reported in the literature, our findings suggest that medical students who feel they are more prepared for exams tend to outperform students who felt less prepared. Purchased resources and the total number of resources used do not seem to have a significant impact on how well students perform on exams. Students who watched lectures online and those who utilized peer-to-peer tutoring services scored higher on exams than students who did not utilize these tools. There was no improvement in exam score seen for students who attended lectures in person. The use of a post-exam survey to evaluate specific resource utilization and exam performance may be helpful for medical school administrations in their selection of supplementary resources for preclinical education. These findings may allow medical schools to modify their curricula and methods of disseminating learning materials based on what is most accessible and feasible for each school. Evaluating resources based on a school’s individual curriculum has the potential to reduce financial burden while reassuring students that they are using validated resources to most effectively prepare for their exams.

## Data Availability

The datasets generated during and/or analyzed during the current study are available from the corresponding author on reasonable request.
